# New Allergens of Relevance in Tropical Regions: The Impact of *Ascaris lumbricoides *Infections

**DOI:** 10.1097/WOX.0b013e3182167e04

**Published:** 2011-05-15

**Authors:** Luis Caraballo, Nathalie Acevedo

**Affiliations:** 1 Institute for Immunological Research, University of Cartagena, Cartagena, Colombia; 2 Foundation for the Development of Medical and Biological Sciences, Cartagena, Colombia

**Keywords:** *Ascaris*, hygiene hypothesis, asthma, ABA-1, Asc l 3

## Abstract

One of the many aspects of the relationships between parasite infections and allergic diseases is the possibility that allergens from parasites enhance the T_H_2 responses, especially IgE production, in allergic diseases such as asthma. In this review we discuss about the allergenic composition of the nematode *Ascaris lumbricoides *and their potential impact on allergy sensitization and asthma pathogenesis and prevalence in populations living in the tropics and naturally exposed to both, mite allergens and helminth infections.

## 

I *Blomia tropicalis *and *Dermatophagoides pteronyssinus *are the most important sources of clinically relevant allergens sensitizing patients with asthma in the tropics, [1-7] although they are also important in some sub tropical areas. Even though several epidemiological and clinical studies show that mite exposure in the tropics is perennial[8-10] and this may induce a sustained and strong IgE response, 2 important points deserve further investigation: first, the high frequency and intensity of the IgE response to mite allergens, [2,11-15] and second, the high prevalence of asthma observed in some urban zones of this region [16-26].

One possibility is that these observations are some of the many consequences of the complex relationships between mite sensitization and soil transmitted helminth infections. In the tropics parasitic infections are still very frequent; helminths cause great morbidity and are one of the main sources of public health problems in underdeveloped countries. In testinal worms, or soil transmitted helminths, infect more than 2 billion people and *Ascaris lumbricoides *alone infects more that 1.2 billion [27,28]. The patterns of infection vary regionally but exposed people living in urban setting suffer mild, intermittent infections by *Ascaris*. Although, in the future, with the improvement of hygiene conditions and lifestyle, parasitic diseases are expected to be eradicated in the tropics, in the current historical moment they are still modifying the pathogenesis and presentation of allergic diseases.

## The Impact of *A. Lumbricoides *Infection (Ascariasis) on the Allergic Responses

There is a large number of epidemiological and experimental works, both in humans and animals, supporting the idea that ascariasis modifies the pathogenesis of asthma and other allergic diseases (reviewed in[29,30]). These investigations have detected that, depending of several factors related to the type of parasites, the host, timing of exposure, infection intensity and the environment, nematode infections may induce either severe immunosuppression or enhancement of the T_H_2 responses. Table 1 shows a list of issues supporting the hypothesis that, in some conditions, ascariasis enhances IgE responses to environmental allergens and allergies.

**Table 1:** Issues Supporting the Hypothesis That Ascariasis Enhances IgE Responses to Environmental Allergens and Allergies

Finding	References
Natural infection is associated with high levels of total and anti-Ascaris IgE responses	[46,68,82,102-107]
In some individuals, natural infection induces IgE-mediated allergic respiratory and cutaneous symptoms	[44,108,109]
In experimental human and animal models, bronchial challenges with Ascaris extract induce asthma symptoms	[45,47,48]
Experimental ascariasis in animals enhances IgE response to bystander antigens	[110-113]
Several epidemiological surveys have found that ascariasis is a risk factor for asthma and atopy	[68,114-119]
IgE responses to Ascaris allergens is more frequent and stronger in mite-sensitized asthmatic patients	[15,82,120,121]

Nematode infections bring together, but in different proportions, immunomodulation and IgE hyper-responsiveness; the latter is also a feature of the allergic responses and depends on the genetic background of the host. However, to understand the mechanisms underlying this particular phenotype, it is also essential to know the antigenic and allergenic composition of both, domestic mites and *Ascaris*, because they do not have the same clinical relevance. The majority of allergens from *D. pteronyssinus *and *B. tropicalis *are already identified, although the clinical relevance, biologic functions and molecular structure of most of them are still under study [6,31-33]. However, most of the allergens from *A. lumbricoides *are unknown, and for a long time, the evaluation of IgE response in humans has been based on the use of the whole parasite extract or preparations from corporal fluids.

## Parasite Infections Associated with Allergic Symptoms

The possibility that nematode allergens have an important role in allergies has been suspected for a long time because there are helminth-infections associated with allergy, IgE-mediated symptoms. The most typical is anisakiasis that induces asthma-like syndrome, urticaria, and anaphylaxis [34-36]. In this case the relationship with allergy symptoms is so evident that some authors consider it more an allergy than an infection[34] and the list of allergens of *Anisakis simplex *officially accepted by the WHO/IUIS Nomenclature Committee is the largest for any parasite. However, before anisakiasis was discovered, clinicians from distinct disciplines have been dealing with hydatidosis, also known as echinococcosis. The rupture of hydatidic cysts that may be located in different places of the body is a well known cause of anaphylaxis, bronchospasm, and urticaria [37-40]. In addition, allergy symptoms associated with the migration of *Strongyloides spp*. and *Toxocara ssp*. are frequently observed in endemic areas [41-43].

Ascariasis is also a recognized cause of allergy symptoms, including *Loeffler's *Syndrome [44-46]. In addition, there are several human and animal experimental models showing the capacity of *Ascaris *antigens to induce parasite-specific IgE response and allergy symptoms (Table 1) [46-49]. However, because the relationship with allergy is not as evident as in anisakiasis, and some reports have demonstrated the immunosuppresor effect of chronic, heavy load infections in rural populations, [50,51] there is the belief that ascariasis only induces immunosuppression. Therefore, to better study this point, it is mandatory to identify those *Ascaris *molecules that induce allergy symptoms, those that generate a protective IgE immune response and those that promote both effects. This will help to elucidate the actual role of these allergens on the pathogenesis of asthma and other allergic diseases and on the associated phenotypes.

Cross reactivity between *Ascaris *and other nematodes, such as *Ancylostoma duodenale, Strongyloides stercoralis, Trichuris trichiuria, Necator americanus*, and *Anisakis simplex*[52-56] has been described and it is possible that it plays a role in the pathogenesis of allergic diseases in the tropics, especially in helminth-helminth coinfections. However, this deserves more investigation because so far it has been mainly studied in relation to the serologic diagnosis of helminthiasis.

## The Allergens of *A. lumbricoides*

A systematic approach for identifying the complete antigens and allergens of *A. lumbricoides *inducing immune responses in humans has not been followed. The antigenic composition of *Ascaris spp*. has been investigated and some molecules (eg, As14, As16, As24, As37, PAS-1) have been analyzed [57,58]. PAS-1 has immunoregulatory properties; As24 and As16 confer protection from migration of *A. suum *larvae through the lungs as demonstrated in experimental vaccination models [58,59]. However, our knowledge on the allergenic composition of *Ascaris *and its clinical impact in humans is still very limited.

There are 2 officially accepted (WHO/IUIS) allergens from this nematode: Asc l 1, also known as ABA-1, and Asc l 3, a tropomyosin. In addition, several IgE binding components have been detected using one dimensional (1D) and two-dimensional polyacrylamide-gel electrophoreses (2D PAGE), [60] and some allergens from other sources, such as *A. simplex *or *Ascaris suum *may be present in *A. lumbricoides*. Furthermore, there are sequence homologies between various *Ascaris *translated-nucleotides entries and recognized allergens, suggesting that the allergenic composition of *A. lumbricoides *is wide-ranging.

**Figure 1:**
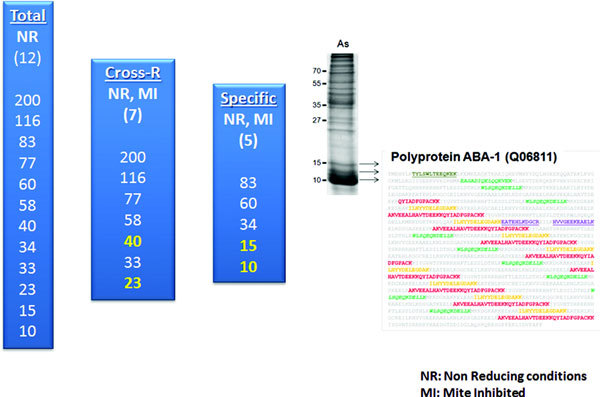
**Among the 12 allergenic components detected with 1D immunoblottings under nonreducing conditions, 7 are cross-reactive, including the 40 and 23 kDa components (Tropomyosin and Glutathione-s-transferase) and 5 are *Ascaris*-specific**. The nematode specific ABA-1 was identified by mass spectrometry in several bands from 10 to 15 kDa.

**Figure 2:**
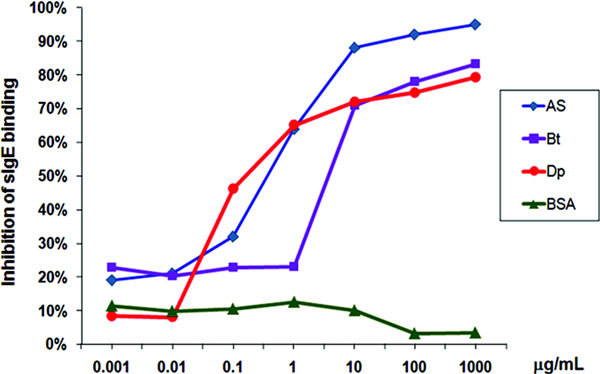
**Ascaris in solid phase, pool of 7 sera from allergic asthmatic patients (reference 60), dilution 1:20**. *B. tropicalis *and *D. pteronyssinus *extracts inhibited 83.3 and 79% of IgE binding to *Ascaris*. Other assay (not shown) detected that *Ascaris *extract inhibited 58.3 and 79.3% of IgE binding to *B. tropicalis *and *D. pteronyssinus*.

Among the 12 allergenic components we have found with 1D immuneblottings under nonreducing conditions, [60] there are 7 that are cross-reactive with mite allergens and 5 that are *Ascaris*-specific (Figure 1). This explains why there is a high degree of cross-reactivity between mite and *Ascaris *extracts (Figure 2) and supports the idea that ascariasis may lead to an enhanced IgE responses to several mite allergens. Tropomyosin and glutathione-s-transferase (GST) are 2 of these cross-reactive allergens.

We recently analyzed, using cross-inhibition ELISA, 1D and 2D immunoblottings and mass spectrometry, the immunochemical properties of *Ascaris *tropomyosin (Asc l 3). Very high allergenic cross-reactivity between the natural Asc l 3 and *B. tropicalis *tropomyosin, Blo t 10, was found using sera from asthmatic patients [60]. These results were confirmed using a recombinant *A. lumbricoides *tropomyosin expressed in a bacterial system [15]. This is the full-length sequence of Asc l 3 and has been classified as Asc l 3.0101. Amino acid sequence identity between mite and *Ascaris *tropomyosins ranges from 73 to 74% and some regions predicted to be IgE binding epitopes in shrimp tropomyosin, were found to be identical in these molecules.

It was also found that IgE antibodies to rAsc l 3 represent a high proportion (~50%) of the total IgE response to an unfractionated parasite extract and there was allergenic equivalence between rAsc l 3 and the native counterpart in the *A. lumbricoides *extract. Furthermore, antitropomyosin IgE antibodies from sensitized subjects reacted against *A. lumbricoides *tropomyosin and induced mediator release in effector cells, both in vivo and in vitro.

ABA-1, although present in other nematodes, is not cross-reactive with any of the *B. tropicalis *or *D. pteronyssinus *allergens, which means that it can be very useful for component-resolved diagnosis of allergic diseases in the tropics. ABA-1 (Asc l 1) is a member of the Nematode Polyprotein Allergen/Antigens [61-63]. Studies support that immune responses (IgG and IgE) to ABA-1 are associated with previous infection and immunity to *Ascaris *[64]. In endemic regions the antibodies isotypes to ABA-1 correlate with the severity of infection, being IgE associated with low infection levels and IgG_4 _or seronegativity with higher susceptibility to the infection [65]. This protein of 15 kDa has only been found in nematodes, has fatty acid binding properties[66] and is synthesized as a polyprotein in gut of the worms and released into the pseudocelomic fluid of the parasite [61,67]. Even though most *Ascaris *allergens have not been characterized, there are data suggesting that Asc l 1 and Asc l 3 play important roles inducing protective antibody immunity and allergy sensitization

## Possible Effects of *Ascaris *Allergens on the Clinical Evolution of Asthma

One potential mechanism to enhance the IgE responses to allergens in asthmatic patients living in the tropics is cross-reactivity (reviewed in[30]). It can act at several points in the evolution of ascariasis or asthma, and the tropical environment provides particular conditions for this effect.

First, there is the possibility of early life coexposure to allergens and antigens from mites and *A. lumbricoides*. The complex interactions elicited by allergenic molecules from different sources acting together on the innate and adaptive immune responses are not yet clearly defined, but one possible outcome is the enhancement of the allergic responses [29]. Early IgE responses to mites and *Ascaris *have been observed in children from tropical regions and some studies have found clinical relevance [68,69].

Second, parasited children in underdeveloped tropical countries receive antihelminth drug therapy during intermittent mass de-worming programs in preschool and school-aged [70]. Because the fundamental socioeconomic causes of the infections are not eliminated, children become reinfected several times with *A. lumbricoides *and this sort of modified secondary immune responses may be boosters of the IgE reactivity against cross-reactive allergens from other sources (eg, mites). In general, antihelminth therapy induces changes of the T_H_2 immune responses; however, the mechanisms involved remain to be elucidated. Repeated treatments significantly increase the production of T_H_2 cytokines, IL5, and IL13 and decrease the production of IL-10 by peripheral blood leukocytes after stimulation with *Ascaris *antigens, although no changes were observed when stimulating with *D. pteronyssinus *and cockroach[71]; however, it was also found that long term periodic treatments in a community with various helminthiasis, including ascariasis, was associated with increase of allergen skin reactivity [72].

In schistosomiasis there is evidence that antihelminthic treatment influences the evolution of several mechanisms of immunity, including increasing of effector T cells proportion and the switch to protective antibody isotypes such as IgE, [73,74] probably because of higher loads of antigens from death parasites, [73,75] and the removal of immunosuppressive parasite products [76]. Reinfections add more possibilities to stimulate memory cells, [77] and some of these mechanisms may work, not only in schistosomiasis but also in other helminthiasis such as ascariasis. For example, it has been reported that treatment of *A. lumbricoides *coinfection may delay HIV-1 disease progression by reducing helminth-induced, IL-10-mediated immunosuppression [78].

Third, as it is well known, mite-allergens exposure is perennial and very intense in the tropics; therefore, in the *Ascaris*-infected population (current or past) susceptible to asthma, this may be other cause of increasing the IgE responses to cross-reactive allergens. It can be speculated that patients predisposed to asthma, with a strong pro-T_H_2 genetic background, early age parasited, suffering several reinfections and permanently exposed to mite allergens probably have a stronger IgE responses to allergens and more severe clinical symptoms.

## The Role of Asc l 1 and Asc l 3 in the Ascariasis/Allergy Relationships

As noted, in humans, the IgE and IgG responses to ABA-1 (Asc l 1) is more related with protection to *A. lumbricoides *infection[64] than with allergy symptoms. However, studies addressed to evaluate directly the allergenic role of this molecule have not been done, and therefore, the possibility that it acts as an allergy-symptoms inducer has not been ruled out. In contrast, tropomyosin is a well recognized invertebrate pan-allergen and Asc l 3 has a high degree of homology and cross-reactivity with mite tropomyosins. Although a role as protective antigen, such as *Onchocerca *tropomyosin[79] is also possible, we have data suggesting that it is associated with allergy symptoms, [15] which may be of epidemiological importance because a high percentage of the population living in the tropics have IgE reactivity to tropomyosins.

Our studies about the IgE responses to ABA-1 and *Ascaris *extract in humans suggest different roles for the allergens of this nematode. For example, in a case-control survey to evaluate the influence of *Ascaris*-specific IgE sensitization on asthma, we found a statistically significant association when the complete *Ascaris *extract was used (Caraballo et al, submitted). However, the significance disappeared when adjusting for total IgE or mite-specific IgE, which confirms the known role of these phenotypes as risk factors for asthma in the tropics. In addition, when ABA-1-specific IgE was investigated, there was no significance at all. Because this search was performed in a large population (421 asthmatics and 620 controls) living in the tropics, it is possible that the weak association detected with the *Ascaris *extract was because of mite-*Ascaris *cross-reactivity among several of their allergenic components. In contrast, ABA-1, which has been previously associated with immunity to *Ascaris *and is not cross-reactive with mite allergens, seems to induce an IgE response not associated with symptoms.

Among the cross-reactive allergens that may underlie the association between Ascaris/extract-specific IgE and asthma, Asc l 3 is a good candidate. Therefore, we further analyzed the role of Asc l 3 as a risk factor for asthma among the subjects with positive anti-*Ascaris*-extract IgE test from the same population. The frequency of sensitization to rAsc l 3 was greater in asthmatics (n = 175) than in controls (n = 170). This result was independent of age and sex. However, when adjusting for covariates such as specific IgE to mites, the significance disappeared, remaining a *P *value of 0.06 [15]. When analyzed as a continuous variable, specific IgE levels to rAsc l 3 were significantly higher in asthmatic patients than in controls. These findings, although not define an independent association of Asc l 3 with asthma, suggest that it may be a risk factor for this disease in the tropics.

Because it is very difficult to define this point by cross-sectional epidemiologic surveys because of the variable origin of the primary sensitization (mites vs *Ascaris*), we are currently evaluating the early IgE responses to purified *Ascaris *and mite allergens in a birth cohort (Risk Factors for asthma and allergic diseases in the tropics, FRAAT) [80]. This will help to better understand the actual role of different antigens and allergens in the pathogenesis of asthma and ascariasis. In addition, animal models analyzing the effects of allergen combinations on sensitization will be also useful.

The possibility of particular roles of ABA-1 and other allergens from *Ascaris *in terms of resistance to the infection and inducing allergic symptoms are also supported by genetic epidemiology studies. There is important evidence that resistance to ascariasis (as evaluated by eggs count in faeces) is probably determined by genes in Chromosome 13q33-34 [81]. Because this region harbor several immune related genes, it was suggested *TNFSF13B *(coding for BAFF cytokine) as a good candidate for explaining the positive linkage results. Therefore, we performed an association study in a population of asthmatics and normal subjects living in the tropics, to investigate the relationships between polymorphisms of 3 genes in that region and the IgE responses to *Ascaris, B. tropicalis*, and *D. pteronyssinus *extracts, the recombinant ABA-1 and asthma [82]. Interestingly, we found association between the IgE response to ABA-1 and the SNP G3980C of *TNFSF13B*. In addition, there was significant association between the variant G299A of *LIG4 *(Ligase IV), and IgE to *Ascaris *extract. However, we found no association between any of the studied markers and the immune responses to mite allergens or asthma. These findings support that antibody responses to ABA-1 is associated with resistance while the IgE responses to other allergens may be associated with allergy. Of course, more studies are needed to dissect the antibody responses against the complete set of *Ascaris *antigens and allergens.

## Why is it Useful for Allergology to Characterize the Allergens of *A. lumbricoides*?

In this review, we argument in pro of the hypothesis that, in the current conditions of socio-economic development of urban areas of tropical countries, ascariasis enhances the IgE responses to mite allergens and, in consequence, influence asthma symptoms. Testing this hypothesis is an important reason to characterize the allergenic components of *Ascaris *and mite extracts, not only because of its potential basic and clinical impact but, in addition, it may explain the high prevalence of asthma in some tropical regions, where viral and bacterial respiratory and gastrointestinal infections are still prevalent, a topic of special interest in regard of the hygiene hypothesis.

## Parasite Infections, Allergy, and the Hygiene Hypothesis

As in its first version, the hygiene hypothesis, raised after some epidemiologic findings, [83,84] predicts that allergic diseases are more frequent in those places where the improvement of hygiene conditions has been really successful, making bacterial and virus infections infrequent in early childhood. The idea is widely accepted and has stimulated several theoretical and experimental approaches to discover basic mechanisms explaining the effects of infections on immune polarization, the inception of IgE sensitization[85,86] and the observed increase of the prevalence of allergic diseases in industrialized countries.

Several factors and conditions influencing the immune system have been proposed to understand how the hygiene hypothesis works. For example, immune deviation to a predominant T_H_1 response because of bacterial and viral infections was, at the beginning, the most obvious explanation; but there is also the suggestion that, instead of infections, there are other elements determining the evolution of the immune responses in children, and, in consequence, acting on the inception of IgE-mediated diseases, among them, the colonization of gut by commensal microbes[87-89] and inhaling cell wall products during infancy [88,90].

An important point is that, according to the immunoregulatory mechanisms proposed to support the hygiene hypothesis, it is expected that allergic diseases have low prevalence in those places where hygiene conditions are poor, and that seem to be true, at least in some particular places, but there are reasons to believe that this is because of chronic helminth infections[50,91-96] and not a result of microbe infections. More interestingly, as it is becoming increasingly known, in several mid- to low-income countries of the tropical zone, the prevalence of asthma and other allergic diseases is high and concur with early bacterial and viral infections [16-26]. And here again, the helminth-infections/allergy relationships may explain why the increasing trend in the prevalence of allergies is more general and not restricted to affluent countries with good hygiene conditions [97,98].

Typically, soil transmitted helminth infections are susceptible to change in frequency when modifying hygiene conditions. During the last decade the immunosuppressive effects of chronic, heavy loads helminth infections have been described in both humans and animals, resulting more evident than any immunomodulatory phenomenon accompanying bacterial or viral infections different from HIV infection. These findings have reinforced the idea that parasite infections have played a major role in controlling the allergic responses; and the lack of this control, because of the improvement of hygiene conditions, has lead to the current figures of allergy prevalence [99,100].

Therefore, the high prevalence of asthma that is currently observed in some urbanized zones of the tropics, where helminth infections, such as ascariasis, are still present but with less intensity than in the past, may be explained, among other factors, as a consequence of the particular historical moment of the ancient and complex relationships between parasites and the immune system: a point where, because of several reasons, the immunostimulating effects of helminths on the IgE responses predominate.

As noted, the type and distribution of parasites, and the frequency, intensity, and immunomodulatory effects of helminth infections, are not the same through the world. In the tropics, both the immunosuppressive and the T_H_2-immunopotentiating effects can be detected, being the latter more frequent at the population level. Then, in this dynamic and changing world, 3 distinct relationships between helminths and the human immune system can be recognized: One with chronic heavy parasite-load infections and mainly immunosuppressive, other of intermittent low parasite-load infections, predominantly IgE-enhancer and associated with urbanization; and a third, with absence of infections, where there is no parasite-derived immunoregulation.

The possible consequences of each of these relationships on the development of allergic diseases have been already analyzed, but 2 additional comments are pertinent. First, a comprehensive study of the human-helminth relationships should include their genetic and evolutionary aspects, which is out of the scope of this review. Here it is just necessary to mention that, because the pathogenesis of ascariasis and other helminth infections, and that of asthma, are highly influenced by genetic factors, these will affect the proportions of individuals that establish any of the proposed relationships in a given population. Second, as suggested by some investigators, the potential effects of other changes associated with urbanization, for instance, differences in diet and lifestyle, physical activity and housing[101] should also be considered.

## Concluding Remarks

One of the multiple faces of the relationships between ascariasis and allergic diseases in tropical environments is specific IgE hyper-responsiveness, mainly induced by *As caris *allergens, mite allergens, and *Ascaris-*mite cross-reactive allergens. As it may affect, not only the evolution of asthma in individual patients, but asthma prevalence at the population level, specific IgE hyper-responsiveness also impacts theoretical aspects of allergology, such as the hygiene hypothesis. Analyzing the various possibilities that may explain these particular host-parasite relationships, demands important basic, clinical, and epidemiological research. The availability of well characterized *A. lumbricoides *and mite allergens will be very helpful.

## End Note

Funded by the Administrative Department of Science, Technology and Innovation (Colciencias-Colombia); Grants 325-2006, 093-2007, and 680-2009.

Part of the data were presented at International Symposium of Molecular Allergology (ISMA), 2010-10-31, Munich.
